# Regression to the mean and alcohol consumption: A cohort study exploring implications for the interpretation of change in control groups in brief intervention trials^☆^

**DOI:** 10.1016/j.drugalcdep.2013.11.017

**Published:** 2014-02-01

**Authors:** Jim McCambridge, Kypros Kypri, Patrick McElduff

**Affiliations:** a Faculty of Public Health and Policy London School of Hygiene and Tropical Medicine, 15-17 Tavistock Place, WC1H 9SH, London; b Centre for Clinical Epidemiology and Biostatistics School of Medicine and Public Health University of Newcastle, Australia

**Keywords:** Regression to the mean, Brief intervention, Alcohol, Student, Research participation

## Abstract

**Background:**

Reductions in drinking among individuals randomised to control groups in brief alcohol intervention trials are common and suggest that asking study participants about their drinking may itself cause them to reduce their consumption. We sought to test the hypothesis that the statistical artefact regression to the mean (RTM) explains part of the reduction in such studies.

**Methods:**

967 participants in a cohort study of alcohol consumption in New Zealand provided data at baseline and again six months later. We use graphical methods and apply thresholds of 8, 12, 16 and 20 in AUDIT scores to explore RTM.

**Results:**

There was a negative association between baseline AUDIT scores and change in AUDIT scores from baseline to six months, which in the absence of bias and confounding, is RTM. Students with lower baseline scores tended to have higher follow-up scores and conversely, those with higher baseline scores tended to have lower follow-up scores. When a threshold score of 8 was used to select a subgroup, the observed mean change was approximately half of that observed without a threshold. The application of higher thresholds produced greater apparent reductions in alcohol consumption.

**Conclusions:**

Part of the reduction seen in the control groups of brief alcohol intervention trials is likely to be due to RTM and the amount of change is likely to be greater as the threshold for entry to the trial increases. Quantification of RTM warrants further study and should assist understanding assessment and other research participation effects.

## 1 Introduction

Like most behaviours, alcohol consumption varies substantially over time (Finney, 2008). Although there are systematic forces shaping alcohol consumption, including its compatibility with other activities, there is also random variation. In longitudinal studies of behaviour, within-subject random variation applies at all timepoints and therefore regression to the mean (RTM; Barnett et al., 2005, Morton and Torgerson, 2005) can operate. RTM refers to the way in which a series of independent observations on a group of individuals will over time approximate the true mean value for that group. Within-subject variability poses an obvious threat to valid inference in longitudinal studies which needs to be controlled, and there are various means available to do so (Skog and Rossow, 2006, Ripatti and Makela, 2008, Gmel et al., 2008). This phenomenon is well recognised and the issues it raises for the study of alcohol or other drug use have been elaborated (Finney, 2008).

Observations which deviate substantially from the true mean are likely to be followed by observations closer to the true mean, which has implications for using thresholds to select individuals for study, e.g. exceeding a given value on a screening test (Barnett et al., 2005). Some people will be selected whose true mean value lies below the threshold, and for whom the observation was unusually high, while others will not be selected whose true mean value lies above the threshold, and for whom the observation was unusually low. If trial eligibility is determined on the basis of total past week consumption, those who celebrated a birthday last week and drank more than usual may be erroneously included, and those who were in hospital may be erroneously excluded.

In brief intervention trials there has been longstanding attention to change in control groups (Bien et al., 1993, Fleming et al., 1997) and to the possibility that aspects of taking part, such as being assessed, may themselves encourage people to think about and reduce their drinking (McCambridge, 2009). Historically this possibility first attracted attention in the alcohol field in treatment studies (Gallen, 1974) and more recently it has featured in brief intervention research (McCambridge and Kypri, 2011). The extent of change over time seen in some treatment trials is striking. This may reflect the natural history of behaviour change among treatment seekers, some of whom decide to cut down or stop drinking before treatment commences. For example, in one study of alcohol dependent women, 44% were abstinent by the time treatment commenced (Epstein et al., 2005). The interpretation of change over time in treatment trials is thus complicated by self-initiated change.

In opportunistic recruitment of non-treatment-seekers to brief intervention trials, change greater than usual variability is also often observed. Control group participants report reducing their drinking by approximately 20% in brief intervention trials (Jenkins et al., 2009, Jenkins et al., 2010), which is larger than the overall between-group differences post intervention (i.e. the intervention effect) in these types of trials (Kaner et al., 2007). For similar reasons, attention to the content and outcomes of control conditions in behavioural intervention trials has also grown recently in other fields (Freedland et al., 2011, De Bruin et al., 2010).

Randomisation safeguards inferences about intervention effects because with large samples RTM is likely to be equivalent across randomised groups. A causal relation between exposure to the intervention and the outcome is only inferred where differences between groups are observed at follow-up (or differences between groups in the extent of change from baseline). Change within an intervention or control group cannot logically be attributed to the intervention or any aspect of the study, in part because of RTM (Finney, 2008). It is not yet known to what extent change over time in alcohol consumption in control groups may be explained by RTM. Degree of change should be expected to vary according to characteristics of the assessment instrument and study design. Quantifying RTM is essential for establishing how far research participation and the procedures involved therein may account for otherwise unexplained change in trials (McCambridge, 2009). Behaviour change caused by research participation itself is important because it may bias intervention effect estimates (McCambridge and Kypri, 2011, McCambridge et al., 2013). The aims of this study are to quantify the contribution of RTM and to consider implications for interpreting findings from brief alcohol intervention trials.

## 2 Methods

We used data from a longitudinal study of alcohol consumption involving students from three New Zealand tertiary education institutions (Kypri et al., 2002a). Students (*n* = 1480) living in halls of residence, and in their first or second year of study (mean age 18.3 years, SD 1.6 years), completed a 12 page pen-and-paper questionnaire anonymously at the start of semester 1, and 967 of them (65%) completed a similar questionnaire in semester 2, six months later. The 967 participants who completed both questionnaires were included in the present study. Questions included the 10-item Alcohol Use Disorders Identification Test (AUDIT), administered in standard form, i.e. without a reference period for items 1–3, a past year reference period for items 4–8, and past year and lifetime response options for items 9 and 10. This screening instrument has been extensively validated with a threshold score of 8 indicating hazardous consumption warranting intervention (Saunders and Baily, 1993, Reinert and Allen, 2007). Research within this population shows that when answering questions 1–3, which concern alcohol consumption, respondents typically reflect on their drinking in the previous 2–3 months (Kypri et al., 2002b). This study population was unselected in relation to their drinking behaviour (all residents were invited to participate), though hazardous drinking was expected to be prevalent (Kypri et al., 2010).

There are formulae for calculating the expected effects of RTM incorporating total variance, between-subject variance, within-subject variance and the correlation between the two (see Barnett et al., 2005 for example). These formulae assume a normal distribution, which rarely applies for alcohol data. We thus use graphical methods as recommended for exploring RTM effects (Barnett et al., 2005). We also apply the conventional threshold score of 8 and quantify the effects of RTM on the AUDIT scores of those who would usually be selected for participation in a brief intervention trial with this criterion (e.g. Kypri et al., 2008b). Subsequently we examine the extent to which using a range of alternative threshold scores (12, 16 and 20) yields different estimates of change. These scores were selected because they have been used previously in decision-making about matching intervention content to severity (Babor and Higgins-Biddle, 2001) and this study is designed to be useful to researchers using the AUDIT.

## 3 Results

**Fig. 1:**
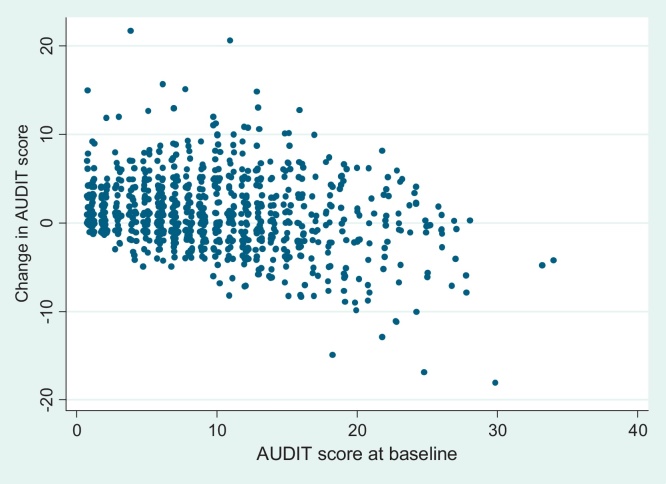
Scatter plot of baseline AUDIT score and change in AUDIT score.

There was a negative association between baseline AUDIT scores and change in AUDIT scores from baseline to six months (spearman rho = −0.17; *p* < 0.001). Students with low scores at baseline tended to have higher scores at follow-up. Conversely, students with high scores at baseline tended to have lower scores at follow-up (Fig. 1). If baseline AUDIT score is used as the eligibility criterion for a brief intervention trial then the mean decrease in AUDIT score from baseline to follow-up increases as the cut point for inclusion increases, even though there is an overall increase of almost one point when the whole study population is considered (see no threshold score in Table 1).

**Table 1:** Mean AUDIT scores and change (95% CI) in AUDIT score.

Population of interest (selection with different baseline AUDIT scores)	Survey		
	Baseline	Follow-up	Change (95% CI)
No threshold	8.87	9.85	0.98 (0.72 to 1.24)
≥8	14.0	14.4	0.43 (0.03 to 0.84)
≥12	16.8	16.6	−0.22 (−0.77 to 0.31)
≥16	20.2	19.0	−1.15 (−1.94 to −0.36)
≥20	23.4	21.4	−1.99 (−3.18 to −0.80)

## 4 Discussion

In this study, in which a cohort of university students’ drinking was assessed at the start of the year and again six months later, the RTM effect increased as the AUDIT cut-off score increased, becoming quite pronounced at the higher thresholds. AUDIT scores increased by one point overall, and among participants who scored 8 or higher at study entry, scores increased by a little under half as much. Thus, RTM as a function of the usual threshold score on this instrument appeared to account for approximately half the observed change over time. It should be noted that any inference regarding the contribution of RTM to the observed change in AUDIT scores is based on the assumption that there were no other influences applying differentially to lighter and heavier drinkers respectively. It is clear from this study, however, that the extent of change over time in a brief intervention control group will depend on the threshold for trial eligibility, with implications also for the interpretation of change in alcohol treatment trials, which typically use higher thresholds for eligibility.

RTM has been somewhat controversial. Many or most high profile statisticians believe this is a real problem that warrants further investigation (e.g. Senn, 1997), whilst others disagree (e.g. Gottman and Rushe, 1993). We used a non-parametric approach, and there are approaches to examining RTM when the data are not normally distributed (for example, Chesher, 1997) but they require assumptions in terms of the distribution of the true value of the measure and also the distribution about the within-person variable, making them more complex than approaches that assume normality (Senn, 1997).

Our approach also assumes follow-up data are missing at random. Loss to follow-up was not obviously biased with respect to alcohol consumption at baseline, as those who did not complete follow-up had similar baseline AUDIT scores to those who were followed up (Kypri et al., 2002a). It remains possible that subsequent drinking behaviour was associated with the likelihood of participation in the follow-up study, with implications for estimates of change over time and RTM. The increase in consumption across the academic year is congruent with the accumulating effects of exposure to heavy drinking among peers (Kypri et al., 2007), very high availability of alcohol (Kypri et al., 2008a) and aggressive promotion (Cousins and Kypri, 2008). It is possible that for at least some of these influences, the effects on lighter drinkers were more pronounced than on heavier drinkers, e.g. peer influences.

The external validity of these findings needs to be carefully considered because the changes we observed are different from those among control group participants in brief intervention trials where reductions over time are typically observed (Bien et al., 1993). Trials evaluating brief interventions specifically to reduce student drinking also typically show large reductions in non-intervention control groups from baseline to the first follow-up timepoint. For example, the well-known trials by Borsari and Carey (2000), Carey et al. (2006), Chiauzzi et al. (2005) and Walters et al. (2009), show reductions of 3–10 standard drinks, or approximately 15–40% of baseline weekly drinking, 1–3 months later at first follow-ups in study populations similar to that used here (though the 6 month follow-up duration and trial recruitment also in later college years should be borne in mind). In these trials, the reductions within the control groups were similar to or larger than the differences between the intervention groups at the same follow-up. It is difficult to draw strong quantitative inferences from direct comparisons, however, due to differences in study designs including selection criteria and in outcome measurement.

Measurement periods are usually shorter for direct measures of alcohol consumption than are reference periods for the AUDIT, and this should mean greater RTM effects when the former are used. The mean increase in drinking seen here among those with AUDIT scores of ≥8 differs strikingly from control group data in the brief intervention trials referred to above, notwithstanding the caveats. Although AUDIT scores are not the same as alcohol consumption, the consumption items accounted for 63% of the overall scores (Kypri et al., 2002a). The increase seen in the present study compared to the reductions found in the brief intervention trials suggests that the behaviour of control groups in these trials do not represent the natural history of student drinking over the course of the academic year, if the data from our longitudinal study are valid. It may be that taking part in brief intervention trials has effects on participant cognitions and behaviour that are distinct from taking part in other longitudinal studies (Kypri et al., 2011).

Making inferences about intervention effects in randomised trials rests on the assumption that there is an additive relationship between intervention effects and research participation effects (McCambridge et al., 2011). This will not be the case where interventions and assessments share the same mechanisms of effect, e.g. altered self-regulation is a plausible candidate for both brief intervention effects and assessment reactivity (Clifford and Maisto, 2000). The implication is that assessment performs some of the work of intervention thus producing a ceiling effect in the form of a statistical interaction between the two (McCambridge, 2013). Bias arising from RTM is protected against by randomisation in trials, which distributes it equivalently between groups with sufficiently large numbers (Finney, 2008), so that intervention effects can be estimated validly in the absence of such interactions. Given how little we appear to know about the nature of change in these studies, this assumption of a lack of interaction deserves further empirical scrutiny.

## Role of funding source

JM is supported by a Wellcome Trust Research Career Development fellowship in Basic Biomedical Science(WT086516MA). There was no specific funding for this study and thus nothing else is declared.

## Contributors

The first two authors had the idea for this study, using a dataset gathered by the second author. The third author undertook all statistical analyses reported here. The first author produced the first draft of this report and led the redrafting, to which all authors contributed. All authors approved the paper for submission.

## Conflict of interest

No conflict declared.
